# Increased Wounding of Southern Right Whale (*Eubalaena australis*) Calves by Kelp Gulls (*Larus dominicanus*) at Península Valdés, Argentina

**DOI:** 10.1371/journal.pone.0139291

**Published:** 2015-10-21

**Authors:** Carina F. Marón, Lucas Beltramino, Matías Di Martino, Andrea Chirife, Jon Seger, Marcela Uhart, Mariano Sironi, Victoria J. Rowntree

**Affiliations:** 1 Department of Biology, University of Utah, Salt Lake City, Utah, United States of America; 2 Instituto de Conservación de Ballenas, Buenos Aires, Argentina; 3 Programa de Monitoreo Sanitario Ballena Franca Austral, Puerto Madryn, Chubut, Argentina; 4 School of Veterinary Medicine, University of California Davis, Davis, California, United States of America; 5 Diversidad Animal II, Universidad Nacional de Córdoba, Córdoba, Argentina; 6 Ocean Alliance/Whale Conservation Institute, Gloucester, Massachusetts, United States of America; New York Institute of Technology College of Osteopathic Medicine, UNITED STATES

## Abstract

At least 626 southern right whale (*Eubalaena australis*) calves died at the Península Valdés calving ground, Argentina, between 2003 and 2014. Intense gull harassment may have contributed to these deaths. In the 1970s, Kelp Gulls (*Larus dominicanus*) began feeding on skin and blubber pecked from the backs of living right whales at Valdés. The frequency of gull attacks has increased dramatically over the last three decades and mother-calf pairs are the primary targets. Pairs attacked by gulls spend less time nursing, resting and playing than pairs not under attack. In successive attacks, gulls open new lesions on the whales’ backs or enlarge preexisting ones. Increased wounding could potentially lead to dehydration, impaired thermoregulation, and energy loss to wound healing. The presence, number and total area of gull-inflicted lesions were assessed using aerial survey photographs of living mother-calf pairs in 1974–2011 (n = 2680) and stranding photographs of dead calves (n = 192) in 2003–2011. The percentage of living mothers and calves with gull lesions increased from an average of 2% in the 1970s to 99% in the 2000s. In the 1980s and 1990s, mothers and calves had roughly equal numbers of lesions (one to five), but by the 2000s, calves had more lesions (nine or more) covering a greater area of their backs compared to their mothers. Living mother-calf pairs and dead calves in Golfo Nuevo had more lesions than those in Golfo San José in the 2000s. The number and area of lesions increased with calf age during the calving season. Intensified Kelp Gull harassment at Península Valdés could be compromising calf health and thereby contributing to the high average rate of calf mortality observed in recent years, but it cannot explain the large year-to-year variance in calf deaths since 2000.

## Introduction

**Fig 1:**
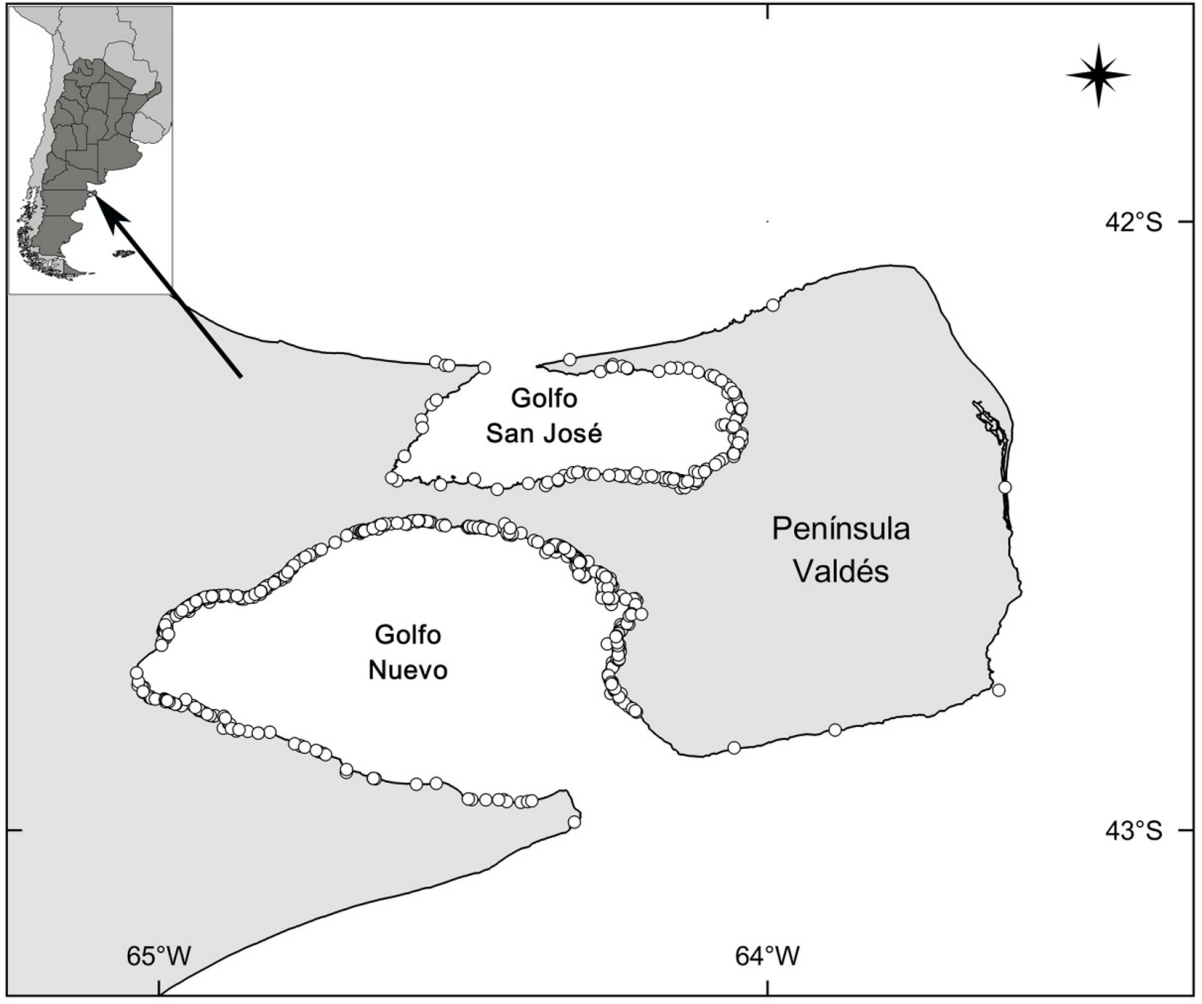
Locations of dead calves at Península Valdés, Argentina. White circles represent the locations of 626 dead calves that were found at Valdés in the period 2003–2014.

An individual's health and reproductive success may be strongly affected by many environmental stressors. In baleen whales, nutritional stress, exposure to algal biotoxins, infectious diseases, and various anthropogenic factors have been shown to influence reproduction [1–9]. Southern right whales (*Eubalaena australis*) usually migrate from their high-latitude feeding grounds in the summer to their low-latitude calving grounds in the winter where females give birth to their calves after a year of gestation [10]. Most calving events usually occur in August for the southern right whale population that calves off Península Valdés, Argentina, [11] and mother-calf pairs usually stay on this calving ground for approximately three months before migrating to their feeding grounds [12]. Females usually calve once every three years [13] and are rarely seen at Valdés in non-calving years [14]. Recently, unusual high calf mortality events have occurred in this population. From 2003 to 2014, at least 626 calves died at Península Valdés with an average of more than 50 deaths per year [15–17] Fig 1. During the previous decade (1993–2002), the average deaths per year had been 8.2, with no more than 14 dead calves detected in any year since 1971. Despite considerable research effort since 2003, the cause(s) of the recent high-mortality events remain undetermined [15,18].

Harassment by Kelp Gulls (*Larus dominicanus*) has been proposed as a potential contributor to the calf deaths [15,18]. Kelp Gulls at Valdés have learned to feed on the skin and blubber of living right whales [19–23]. During an attack, a gull lands on a whale’s back and gouges out skin, either opening a new lesion or enlarging a preexisting one [20,21]. During successive attacks, gulls widen and/or dig more deeply into the dermal and subdermal layers of skin, exposing blubber and creating lesions of various sizes and depths. Kelp-Gull attacks usually occur when whales are resting or surfacing to breathe and their backs are fully or partially exposed to the air [20].

Attacks were first reported at Península Valdés in the 1970s when Kelp Gulls occasionally picked sloughed skin from the water or the whales’ backs [19]. In the 1980s, Thomas [20] described gull attacks as “parasitic” with gulls gashing the skin and underlying blubber of whales in Golfo San José. Mothers were the predominant target, and calves were rarely attacked. In the mid-1990s, both mothers and calves that were followed continuously for an hour were attacked in both gulfs of the peninsula (Golfo San José and Golfo Nuevo) at an average frequency of 17% of the total observation intervals recorded [21]. By 2005–2012, gull attack rates escalated to 24% in both gulfs, and most attacks (69%) were aimed at calves [23]. In 2012, some dead calves that stranded on the beaches had 25 or more gull-attack lesions on their backs [18]. Kelp Gull harassment has not been observed in any other cetacean population, except for two isolated cases recorded in southern right whales off Brazil in the 1990s [24].

Gull harassment negatively affects the behavior, energy expenditure and probably the body condition of mothers and their calves. In 1995, mother-calf pairs harassed by gulls more than tripled the time spent in high-energy activities compared to those that were not attacked. Pairs swam at medium or fast speeds to flee gull attacks, reducing time spent in low-energy activities (e.g., resting and traveling slowly) by 25% [21]. Low-energy activities are the predominant behaviors of lactating right whale mothers at Valdés when not disturbed by gulls [12, 20,21]. Lactating mothers on the calving grounds are fasting and relying on blubber reserves for self-maintenance and nursing. Mothers cannot recover energy reserves spent fleeing gull attacks because food is available only occasionally at Valdés during the calving season [25–27]. Calf survivorship may be compromised if mothers and calves invest more energy and time fleeing Kelp Gulls than resting and nursing. Constant gull harassment could also increase the metabolic stress of mother-calf pairs while on the calving ground.

In 2013, a workshop was convened to consider possible causes of the high calf mortality at Valdés. The specialists present concluded that Kelp Gull harassment is probably a major stress factor for the whales: “the physical injury of extensive gull lesions can compromise the integrity and impermeability of the whale’s surface layers and lead to dehydration, loss of thermoregulatory capacity, and increased energy outlay to wound healing and metabolic stasis” [18]. Thus, gull attacks could negatively affect body condition of mother-calf pairs during a critical period when calves depend on maternal milk for growth and survival.

The intensity of gull attacks on an individual whale can be estimated from the number and size of lesions on its back. Here we focus on the history of wounding in living and dead calves at Península Valdés over the last four decades. We ask specifically whether the increase in wounding rates has been similar for living calves and their mothers, and whether living and dead calves suffered more wounding in years of high calf mortality during the period 2003–2011. We also look for effects of calf age (using body length as a proxy) and location (Golfo San José *versus* Golfo Nuevo) on the intensity of gull attacks.

## Materials and Methods

### Presence and number of lesions on living mother-calf pairs

To determine the presence, number and size of lesions on living mother-calf pairs, we used photographs taken during annual aerial surveys at Península Valdés in 1974–1979, 1982–1990, 1993, 1995, 1996, 1999, 2000 and 2002–2011 (1971–73 and 1980–81 were excluded because of poor photo quality or incomplete survey coverage). Photographs of southern right whales are used to identify individuals (especially adults and juveniles) through the distinctive callosity patterns on their heads and pigmentation patterns on their backs [28]. Only photo-identified mothers were selected to avoid recording a mother and her calf more than once in a given year. Mothers resighted within two to thirty-seven years were included in the analysis because most lesions disappear within two to four years [21]. Most calves included in the study were not individually identified because their callosity patterns are not fully developed in their first three months of life and therefore few of them (females) were resighted years later accompanied by a calf. Calves examined in the study were calves of the season and were mostly older than a month and few newborns since most aerial surveys are done in September-October (around the time of peak whale abundance; [14,29]), one or two months after the majority of calving events occur [11]. Locations of sightings at Valdés were also recorded (Golfo Nuevo, Golfo San José and Outer Coast, Fig 1) during the aerial surveys [14].

The presence of lesions was scored categorically for each mother and calf as follows: “yes” if the whale had at least one lesion or “no” if it had no lesions. Lesions were scored only if the center of the whale’s back was clearly visible (above water or under water). Whales were scored as having zero lesions (“no”) only if the center of the back and ≥70% of its total area were clearly visible. If the whale had lesions (“yes”) and its back was ≥50% visible, then the total number of lesions was recorded and their shape, placement and size were drawn on a silhouette (S1 Fig). We did not count the number of lesions if there were one or more ("yes") but less than 50% of the back was visible. Presence (or absence) of lesions could not be scored in 25% of all right whale mothers photo-identified in 1974–2011 due to their position (underwater, rolling) or poor photo quality. Lesions in calves could not be determined in 41% of all cases due to their position (underwater, hiding below their mothers, nursing, rolling or playing) or poor photo quality.

### Presence and number of lesions on dead calves

To determine the presence, number and size of lesions on dead calves, we used photographs taken during necropsies of calves that died and stranded at Península Valdés in 2003–2011. Although dead calves were in various states of decomposition, only photographs of fresh dead calves without major skin damage (minimal detached skin or damage by scavengers) were selected for this analysis [30]. Necropsies were conducted by the Southern Right Whale Health Monitoring Program (SRWHMP) annually throughout the season (June to December). Necropsied calves varied in length (3 to 9 m), age (newborns to calves less than four months old) and stranding location (Golfo Nuevo, Golfo San José and Outer Coast) with most recorded in the gulfs [16,17,30].

Presence of lesions was determined as described above for living whales: “yes” if the dead calf had lesions or “no” if it had no lesions. We counted and drew lesions on dead calves following the same procedures described above for living mother-calf pairs (backs ≥50% clearly visible and no major skin damage). Presence of gull-inflicted lesions could not be determined in 56% of all recorded calves (n = 438) that died in 2003–2011 because they were upside down or in advanced states of decay (no skin) or the photographs were of poor quality.

### Sizes of lesions on living and dead whales

**Fig 2:**
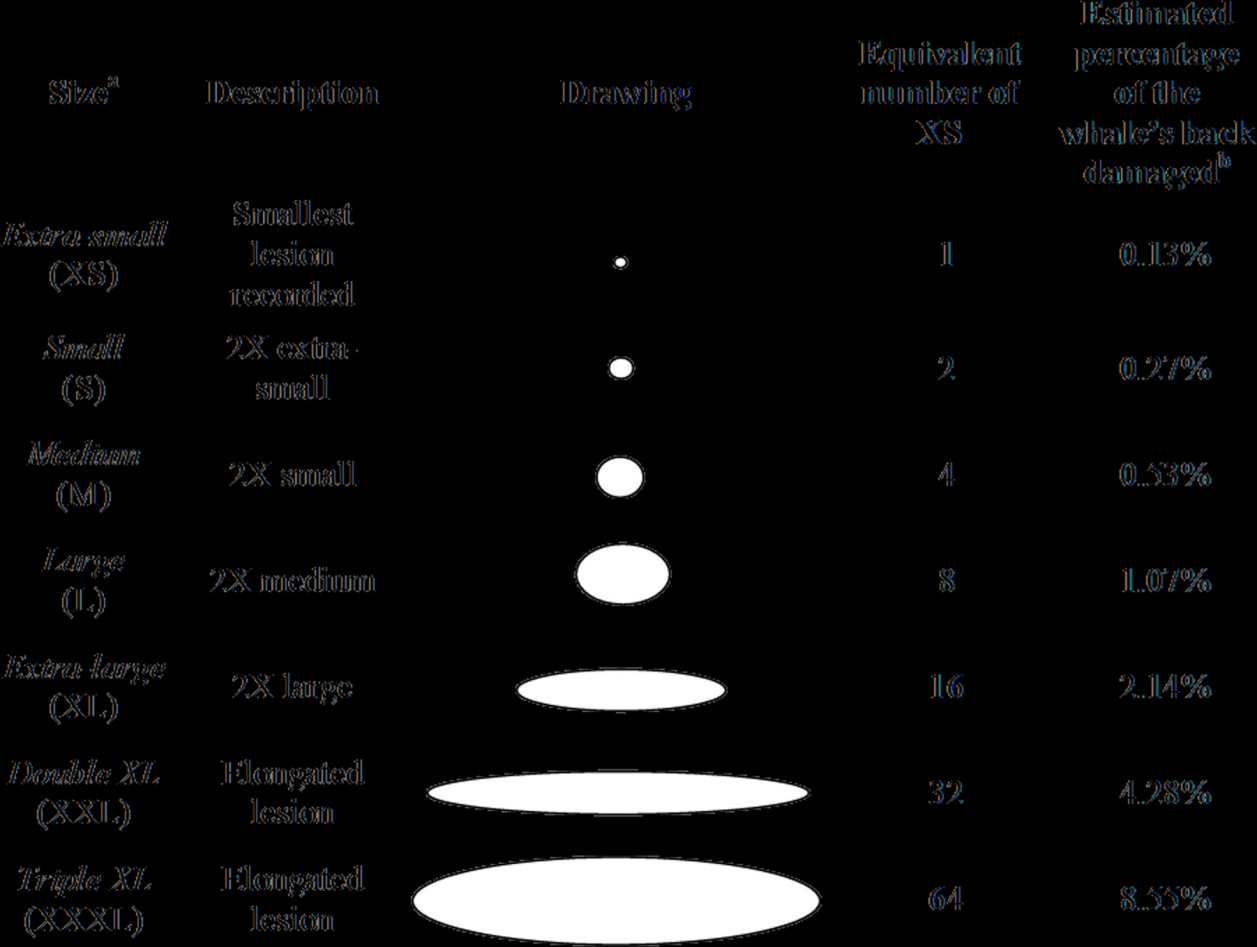
Sizes of gull lesions on southern right whales and their equivalent number of extra-small lesions. ^a^Abbreviations: Extra-small (XS), Small (S), Medium (M), Large (L), Extra-large (XL), Double XL (XXL) and Triple XL (XXXL). ^b^The total back area or TBA (100%) available for scoring extended from the fat roll (behind the blowholes) to the beginning of the tail stock and down the sides to the region of the back that is above the water or clearly visible through the water when the whale is close to the surface (see S1 Fig). See methods for a complete explanation.

**Fig 3:**
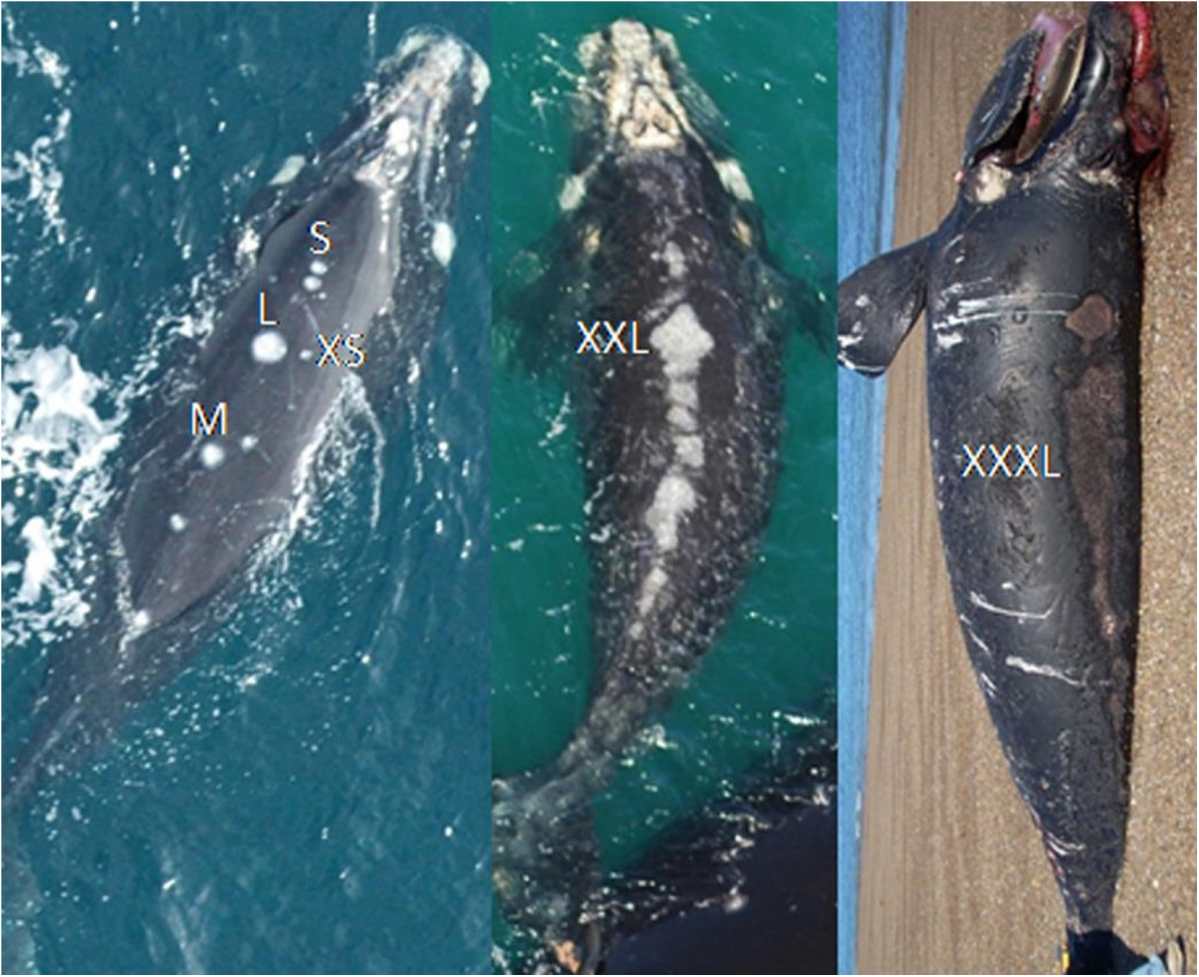
Size of gull lesions. Sizes are shown on the backs of two living (left and middle) and a dead calf (right) photographed at Península Valdés. Each size is indicated by its abbreviation. Abbreviations: Extra-small (XS), Small (S), Medium (M), Large (L), Extra-large (XL) and Triple XL (XXXL). Photo credit John Atkinson and SRWHMP.

We drew lesions in proportion to the size of the whale’s back using a whale silhouette (S1 Fig). Each lesion was assigned to one of seven size categories: extra-small (XS), small (S), medium (M), large (L), extra-large (XL), double XL (XXL) and triple XL (XXXL) (Fig 2). The smallest lesions, XS, represent the earliest stages of a gull-inflicted lesion, when the skin is pecked enough to make a small amount of blubber visible (Fig 3). The absolute sizes of XS and larger lesions are different for mothers and calves, because they are drawn on the silhouettes in proportion to the size of the individual. Thus the size categories represent relative wound sizes, with XS lesions averaging roughly 1/800 of the area of the back, S averaging roughly 1/400, and so on in a doubling series (Fig 2). The elongated and continuous XXL and XXXL lesions are formed by the merger of adjacent lesions (Fig 3). XXL lesions are typically less than half as long as the back, while XXXL lesions typically extend along more than half of the length of the back.

### Area of lesions on living and dead whales

Because a whale with three XS lesions is much less damaged than a whale with three XL lesions, we combined the size and total number of lesions to estimate the overall area of lesions (or wounded area) using the XS lesions as the basic unit of measure (see Fig 2). First, each lesion larger than XS was converted into its equivalent number of XS lesions (see Fig 2). Then the number of XS-lesion-equivalents was added to represent the total area of lesions. For example, a whale that had one XS lesion and one M lesion (number of lesions = 2) was considered to have a total area of five XS lesions because one M lesion is roughly equivalent to four XS lesions.

### Percentage of back damaged on living and dead whales

To determine the percentage of the whale’s back that is damaged by lesions, we used ImageJ [31] to analyze 15 living calves (1974–2011) that had >80% of their backs clearly visible above water. Using the same whale silhouette to draw the lesions (S1 Fig), we drew an oval representing the total back area (TBA) that extends from the fat roll (immediately behind the blowholes) to the beginning of the tail stock and laterally to the "shoulders" (dotted line, S1 Fig). For each calf, we calculated the TBA in pixels which represents 100% of its back. Then we calculated in pixels the total area of all lesions of different sizes (solid lines) and divided it by the TBA to estimate the percentage of damage to the back. Finally, to establish a scaling between XS-equivalents and the percentage of the whale’s back area that is damaged, we calculated the area in pixels of all the XS lesions present on all 15 calves. The average pixel area of all XS lesions was divided by TBA to estimate the percentage of the back damaged by one XS lesion. We then multiplied that percentage by the numbers of XS-equivalents to estimate the standard conversion factors summarized in Fig 2.

### Changes in the number and area of lesions with calf age

Calf length can be used to estimate calf age [11,16,32,33]. We used calf length to ask whether lesion numbers and areas increased with calf age in dead and living calves. Although calving occurs from June to November [11,34], most calves are born in August at an average length of 5.5 meters and grow as much as three meters [11] during their three months on the calving ground [12,35]. However, some calves are shorter than six meters when born, and they predominate among those that died early in the calving season (before October 1) [11] (Table 1). Conversely, calves larger than six meters typically die late in the season (on or after 1 October) [16] (Table 1). We considered dead calves <6 meters to be "small" and those ≥6 meters to be "large". Dead calf lengths were determined with a tape measure as a straight line from snout to fluke notch and were recorded by the SRWHMP during necropsies of dead calves following standard procedures [36,37]. Data for gull-lesion analysis were collected at Valdés in 2003–2011.

**Table 1:** Percent of small (<6 m) and large (% ≥6 m) dead calves by month (n = 569).

Length (m)	June (n = 3)	July (n = 30)	August (n = 142)	September (n = 153)	October (n = 149)	November (n = 75)	December (n = 17)
% <6 m	100	86.67	73.24	63.40	39.60	30.67	29.41
% ≥6 m	0	13.33	26.76	36.60	60.40	69.33	70.59

Calf lengths were measured in meters as the straight-line distance from snout-tip to fluke notch. Data were collected at Península Valdés in 2003–2014 (note that data for gull-lesion analysis was collected only in 2003–2011).

Living calves off South Africa average 40% of their mothers’ lengths at the beginning of the calving season (late July) and increase to 51% of their mothers’ lengths later in the season (mid-October, [33]). Because length measurements were not available for living calves at Península Valdés, we used the calf/mother body length ratio to examine differences in wounding patterns in young *versus* older calves. Living calves less than 50% their mother’s length were considered to be “small” and those greater than 50% of their mother’s length were considered to be “large”. Calf length estimates were made from aerial survey photographs in the ‘90s and 2000s (1993, 1995, 1996, 1999, 2000, 2002–2011).

### Changes in the number and area of lesions in high- and low- mortality years

We asked whether calves (living and dead) in high-mortality years had lesions covering larger areas than calves of similar length in low-mortality years. We defined high-mortality and low-mortality years in the 2000s following Rowntree et al. [16]. Briefly, in low-mortality years (2004 and 2006) the number of dead calves was not significantly greater than expected based on the population's long-term growth rate, while in high-mortality years (2005, 2007–2011) the number of dead calves was significantly greater than expected.

### Statistical analyses

R software version 3.1.1 [38] was used for all statistical analyses. Contingency chi-square tests and one-way ANOVAs were used to determine changes in the intensity of wounding in living mother-calf pairs photographed in aerial surveys from 1974 to 2011. Specifically, the presence, number and area of lesions on living pairs were compared among decades, gulfs (Golfo Nuevo and Golfo San José) and calf lengths (small and large). Living mothers and calves sighted on the Outer Coast were excluded when comparing number and area of lesions per gulf because few mother-calf pairs have been recorded there since the 1980s [29]. However, living mothers and calves sighted on the Outer Coast were included in other comparisons. Additionally, the numbers and areas of lesions were compared in living calves in high- *versus* low-mortality years controlling for calf length (factor variable: “small” versus “large” calves).

Linear regressions and Kendall's rank correlations were used to evaluate changes in the intensity of wounding in dead calves photographed during necropsies from 2003 to 2011. Specifically, the numbers and areas of lesions were compared in dead calves among gulfs (excluding the Outer Coast), in high- *versus* low-mortality years, and in small *versus* large calves. We adjusted by calf length (using the numerical measurements) when comparing lesions among gulfs and mortality levels.

## Results

### Increased presence of lesions on living mother-calf pairs

**Fig 4:**
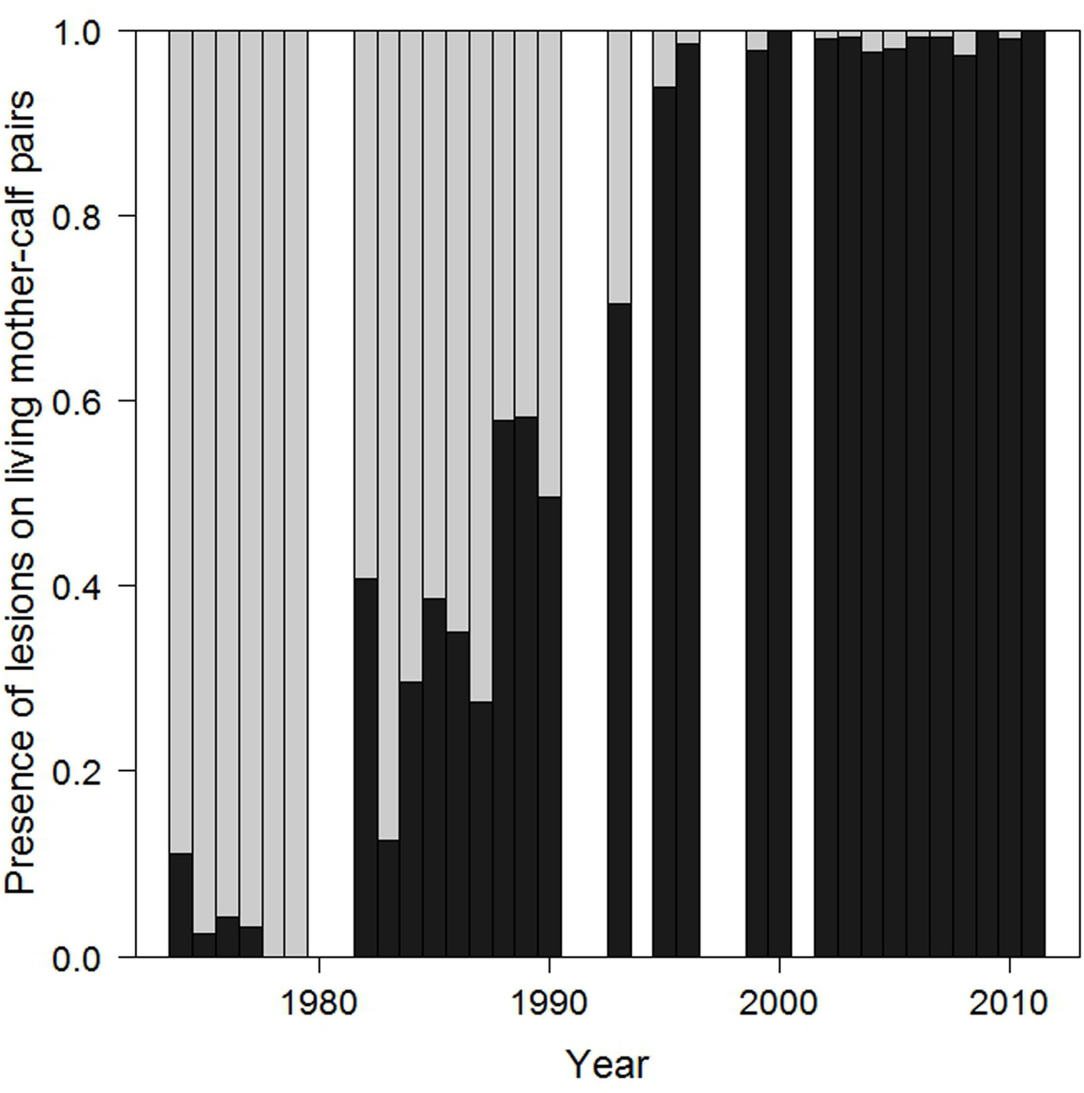
Presence of lesions on living mother-calf pairs in 1974–2011. Light grey bars indicate the proportion of whales without lesions and dark grey bars indicate the proportion of whales with lesions. White columns indicate years excluded from the study.

The presence of gull lesions was assessed in 2680 living whales (1527 mothers and 1153 calves) photographed during aerial surveys in 1974–2011. The proportions of mothers and calves with lesions increased through the four decades (Fig 4, Table 2). Gull-inflicted lesions were rarely seen on mother-calf pairs in the 1970s (2% of pairs examined) but became frequent in the 80s (36%), continued to increase in the 90s (84%) and became nearly universal in the 2000s (99%) (Table 2). These differences are all highly significant (chi-square tests on pairs of decades, or on the whole table).

**Table 2:** Percent of living mother-calf pairs and dead calves with and without lesions by decade.

Decade	Live mothers and calves without lesions	Live mothers and calves with lesions	Dead calves without lesions	Dead calves with lesions
1970s	**97.9%** (n = 231)	**2.1%** (n = 5)	NA	NA
1980s	**61.6%** (n = 297)	**38.4%** (n = 185)	NA	NA
1990s	**16.4%** (n = 105)	**83.6%** (n = 536)	NA	NA
2000s	**1.1%** (n = 14)	**98.9%** (n = 1307)	**43.7%** (n = 84)	**56.3%** (n = 108)

Sample size is indicated between parentheses. Data were collected from aerial survey photographs of living mother-calf pairs in the period 1974–2011 and necropsy photographs of dead calves in the period 2003–2011. NA: no data available.

### Increased numbers and areas of lesions on living mother-calf pairs

**Fig 5:**
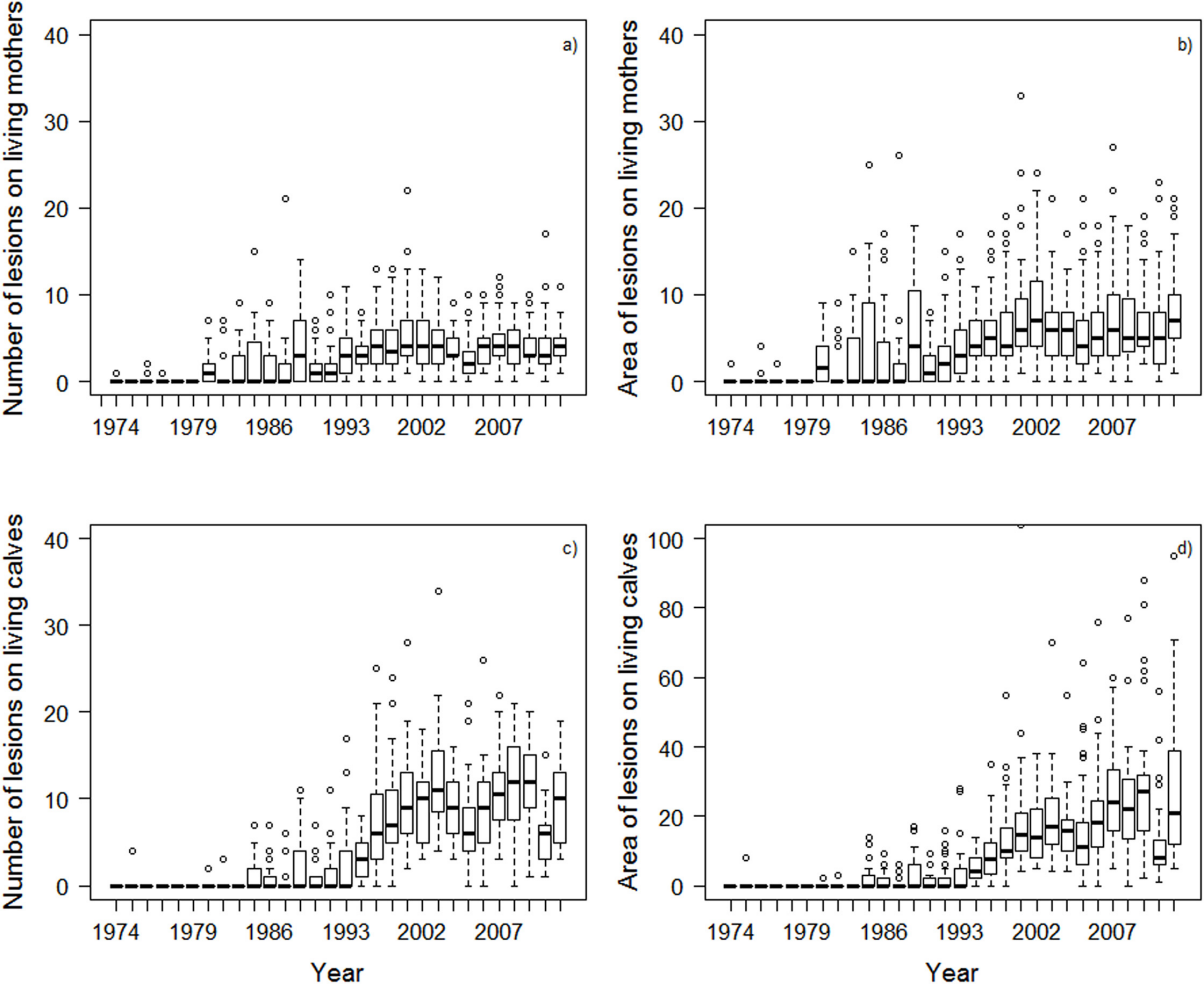
Number and area of lesions on living mothers (a, b) and calves (c, d) in 1974–2011. Median values (thick black line), minimum and maximum (whiskers), first and third quartiles (thin black lines), and outliers (open circles) are shown in the figure. Note the different scale for the y axis in the area of lesions on calves (d). To avoid excessively expanding the y-axis in Fig 5d, two extreme values (y = 104 and y = 147 XS lesions) were excluded.

The numbers and areas of lesions on living mothers and calves were highly variable among years (Fig 5A, 5B, 5C and 5D) and decades (Table 3) with a tendency to increase over time. However, relatively more lesions were recorded on calves in the 2000s than on mothers (Table 3). Typical calves had no lesions in the 70s and 80s, but an average of around nine lesions in the 2000s (Table 3). The numbers of lesions on mothers increased from an average of zero in the 70s to two in the 80s and three or four in the 90s and 2000s. The average area of lesions also increased from the 70s to the 2000s, from zero to twenty XS-equivalents in calves and zero to seven XS-equivalents in mothers. The largest numbers ever recorded were 22 on a mother and 34 on a calf, and the largest areas were 44 XS-equivalents on a mother and 147 XS-equivalents on a calf. A total area of 147 XS lesions on a calf is roughly 19% of its back (Fig 2).

**Table 3:** Wounding of living mothers, living calves and dead calves by decade (1970s-2000s).

Decade	Living mothers		Living calves		Dead calves	
	Mean number	Mean area	Mean number	Mean area	Mean number	Mean area
1970s	0.03±0.22 (n = 143)	0.06±0.41 (n = 143)	0.04±0.42 (n = 92)	0.09±0.83 (n = 92)	NA	NA
1980s	1.80±2.85 (n = 282)	2.79±4.41 (n = 282)	0.90±2.08 (n = 198)	1.46±3.35 (n = 198)	NA	NA
1990s	3.49±2.96 (n = 344)	4.75±3.96 (n = 344)	5.36±5.35 (n = 244)	8.24±12.22 (n = 244)	NA	NA
2000s	4.04±2.67 (n = 592)	6.65±5.10 (n = 592)	9.19±5.04 (n = 390)	19.61±15.71 (n = 390)	3.54±4.42 (n = 186)	9.75±13.94 (n = 177)

Mean and standard deviation of number and area of lesions are shown for whales sighted over four decades of study. All means were significantly different among decades for living mothers and calves (≤0.001, ANOVA) with the exception of living calves in the ‘70s and ‘80s. Sample size is indicated between parentheses. Data were collected using aerial photographs from 1974 to 2011 for living mother-calf pairs and necropsy photographs from 2003 to 2011 for dead calves. NA: no data available.

**Fig 6:**
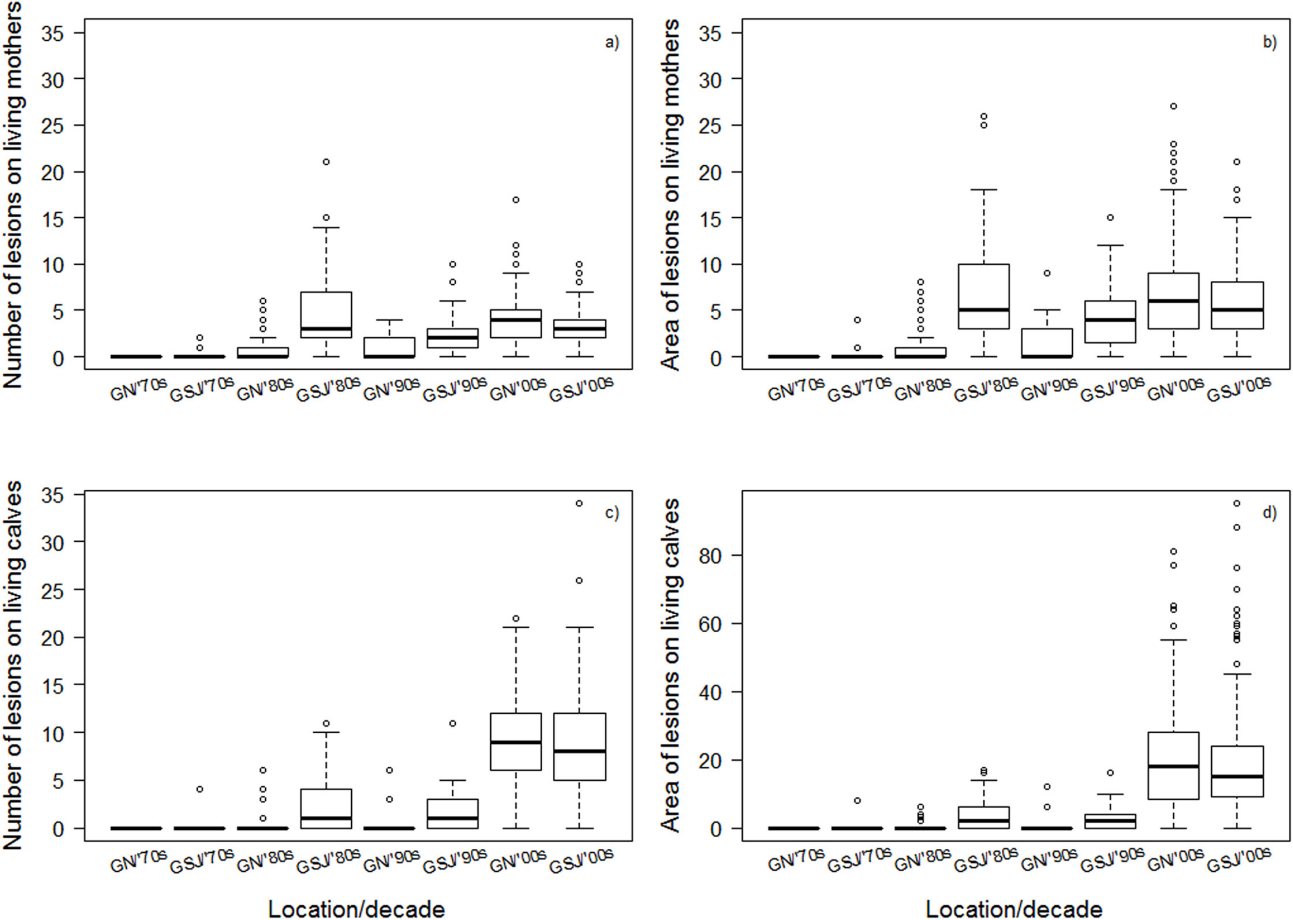
Number of lesions on living mothers (a, b) and calves (c, d) by decade and location (GN: Golfo Nuevo, GSJ: Golfo San José). Median values (thick black line), minimum and maximum (whiskers), first and third quartiles (thin black lines), and outliers (open circles) are shown in the figure.

In the 2000s, the gulf with more wounding changed from Golfo San José to Golfo Nuevo (Fig 6A, 6B, 6C and 6D). In the 70s, 80s and 90s, mothers and calves surveyed in Golfo Nuevo had significantly fewer lesions and smaller wounded areas than those in Golfo San José (ANOVA, number and area of lesions in mothers: all *p* -values < 0.001, number and area of lesions in calves: all *p* -values ≤ 0.01) (Fig 6A, 6B, 6C and 6D, Table A in S1 File). But by the 2000s, mothers surveyed in Golfo Nuevo had more and larger lesions (ANOVA, *p* = 0.01); calves showed the same trend, but it was not significant (ANOVA, *p* = 0.70) (Fig 6A, 6B, 6C and 6D, and 6B, Table A in S1 File).

Both calf length relative to mother and number of lesions were determined in 565 living calves photographed from 1993 to 2011, 87% of which were large calves. Large living calves had more lesions (mean ± SD, 8.61 ± 5.33) than small calves (7.07 ± 4.65) (ANOVA, *p* = 0.02), but their area of lesions was not significantly larger (ANOVA, *p* = 0.17). However, both the number and wounded area were significantly larger in large living calves if the comparison was limited to calves within the 2000s (2003–2011) (ANOVA, number of lesions: *p* < 0.001, lesion area: *p* = 0.01). Although the numbers of lesions were higher in low- (n = 88) than in high- (n = 238) mortality years for calves of similar length (regression, number of lesions *p* = 0.002), there were no significant differences in the area of lesions among low- and high-mortality years (regression, area of lesions: *p* = 0.74).

### Increased Presence, Numbers and Areas of Lesions on Dead Calves

Presence of lesions was assessed in 192 dead calves photographed during necropsies from 2003 to 2011 at Valdés (Table 2). Similar percentages of dead calves were found with gull-inflicted lesions (56.3%) and without them (43.7%). Compared to living calves, number (n = 186) and area (n = 177) of lesions were determined in fewer dead calves because of poor photo quality, back exposure or skin decay. The average number of lesions on dead calves was four (range: zero to 21) and the average wounded area was 10 XS-equivalents (range: zero to 79) (Table 3). An area of 79 XS lesions on calves is roughly 10% of the back damaged by gull lesions (Fig 2).

**Fig 7:**
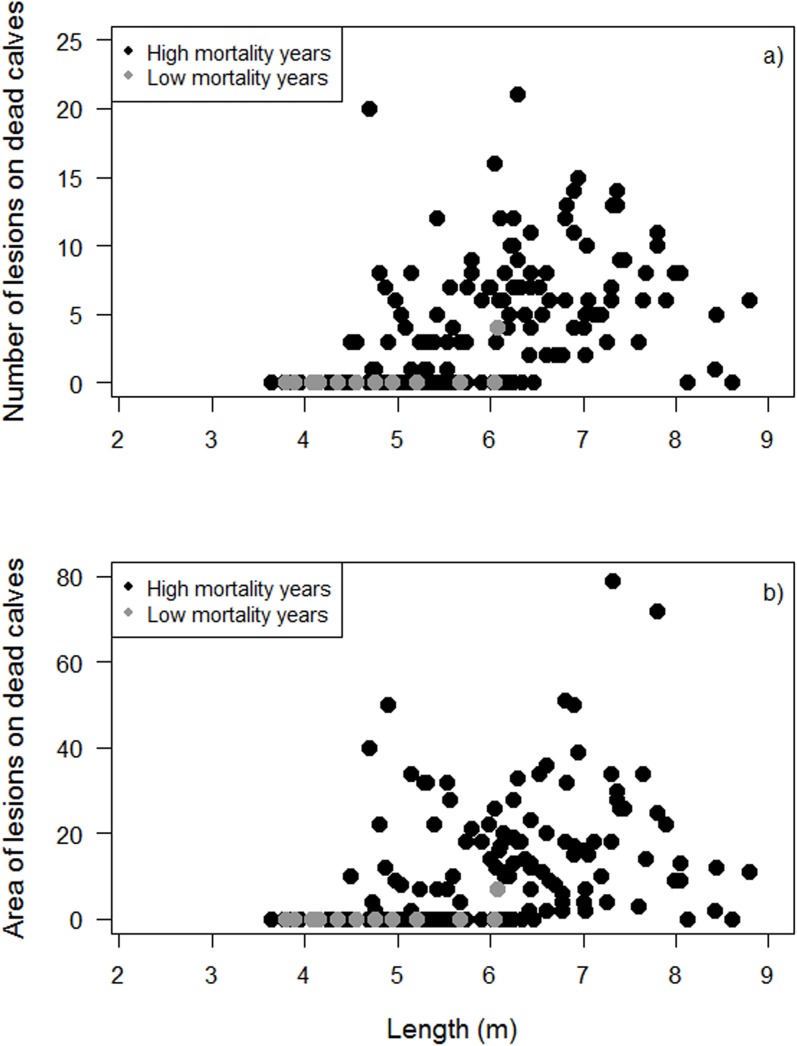
Numbers (a) and areas (b) of lesions by dead calf length in the period 2003–2011. Calves’ length ranged from 3.64 to 8.80 meters. Grey dots indicate calves that died in low-mortality years (n = 12) and black dots indicate calves that died in high-mortality years (n = 174). Kendall's rank correlation, *p* < 0.001.

Both calf length and number of lesions were determined in 181 dead calves that stranded at Valdés in 2003–2011, 44% of which were large calves. The lengths ranged from 3.64 to 8.80 m. Both lesion number and lesion area increased with calf length (Fig 7A and 7B) (Kendall's rank correlation; number of lesions *p* < 0.001; area of lesions *p* < 0.001).

**Fig 8:**
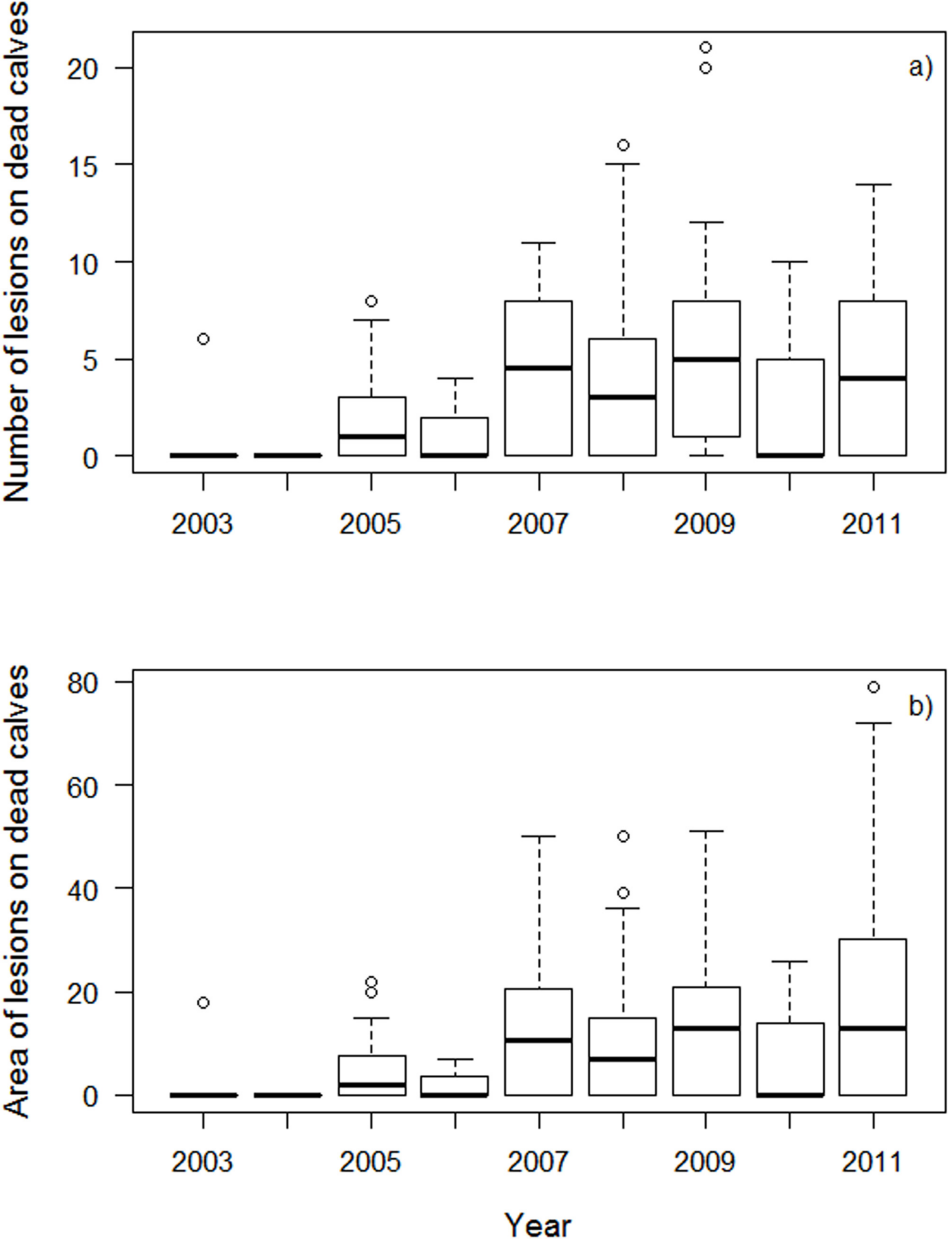
Number (a) and area (b) of lesions on dead calves in the period 2003–2011. Median values (thick black line), minimum and maximum (whiskers), first and third quartiles (thin black lines), and outliers (open circles) are shown in the figure. Low-mortality years: 2004 and 2006, high-mortality years: 2003, 2005, 2007–2011.

Numbers and areas of lesions on dead calves increased from 2003 to 2011 (Fig 8A and 8B). However, adjusting by calf length, there were no significant differences in the number or area of lesions among low- (n = 12) and high- (n = 174) mortality years (Fig 7A and 7B) (regression, number of lesions *p* = 0.23, area of lesions: *p* = 0.26). When controlling for calf length, dead calves that stranded in Golfo Nuevo (n = 139) had larger numbers and wounded areas compared to those that stranded in Golfo San José (n = 46) in 2003–2011 (regression, number of lesions *p* = 0.05, area of lesions *p* = 0.02, Table B in S1 File).

## Discussion

Almost all known observations of gull harassing right whales have have ocurred at Península Valdés. Thus gull harassment might plausibly contribute to the recent high calf mortality events, which are also unique to this area. However, the impact of gull-inflicted lesions on whales has not been fully characterized. Here, we have quantified the increased wounding of mothers and calves over four decades of study. We find that the first pairs documented to have gull-inflicted lesions were photographed in the 1970s and that calves have recently become the main targets of attacks.

Increasing proportions of right-whale mother-calf pairs have gull-inflicted lesions on their backs at Península Valdés. In the 1970s, most living mother-calf pairs were lesion-free. By the 1980s, around a third of pairs carried lesions. In the 1990s, the proportion of pairs with lesions increased significantly and by the 2000s, almost every mother-calf pair had lesions. This finding, from retrospective analysis of aerial survey photographs, supports behavioral studies that find higher frequencies of gull attacks in the 2000s than in previous decades [23,39].

The increased incidence of gull-inflicted lesions on calves as compared to their mothers has not previously been noted. While the number of lesions on living mothers has remained nearly the same with an average of two to four lesions through the 80s, 90s and 2000s, the number of lesions on living calves has greatly increased from an average of one lesion in the 80s to nine lesions in the 2000s. In addition, the area of lesions on living calves has increased dramatically from an equivalent of two extra-small lesions in the 80s to twenty extra-small lesions in the 2000s which is three times the damaged (relative) area on living mothers in the same years. The largest area of lesions recorded for a living calf covered 19% of its back.

Mothers appear to have learned how to defend themselves. The lack of change in number and area of lesions on mothers may be explained by their adopting a variety of resting and breathing postures that help them evade gull attacks [20,39]. Until the late 1980s, mothers were often seen resting at the surface (even for an hour or more) and their backs dried out exposing peeling skin that was pecked off by Kelp Gulls [20]. However, since the mid-1990s, mothers with dry backs are rarely seen because they have adopted avoidance postures such as the "galleon". In the galleon posture (or “crocodiling” according to Thomas [20]), mothers arch their backs exposing only their heads and tail flukes above water. The galleon posture was first documented in 1984 [20] and has spread through the adult population [39].

Unlike mothers, calves have not developed as many avoidance postures and may be more vulnerable to gull attacks. They have shorter backs (compared to adults and juveniles) and may therefore find it difficult to arch their bodies deeply enough below the surface. Perhaps more fundamentally, calves have never experienced Kelp-Gull attacks, while their mothers have had many seasons on the Valdés calving ground to learn about them. Calves surface to breathe much more frequently than adults [23], so they are exposed more often than older whales. Since the 2000s, some calves (and also mothers) have learned to surface at an oblique angle to blow while keeping their backs under water (“oblique breathing” [40]). Oblique breathing may minimize the impact of gull attacks but could also be energetically costly for newborns or young calves [40]. Thus avoiding gull attacks seems likely to be more energetically costly for calves (e.g., swimming away at fast speeds, [21], or performing oblique breathing, [40]) than for mothers, in a period when most of their energy is needed for growth, development and play [12].

Dead calves did not have larger lesions in high-mortality years than in low- mortality years in the period 2003–2011 (Fig 7A and 7B), nor did living calves (Fig 6C and 6D). Furthermore, living calves had more lesions in low-mortality years for the same time period. Thus the dramatic year-to-year variation in calf mortality rates during the 2000s is not explained by the apparently modest year-to-year variation in gull-lesion rates. This implies that other, still unidentified factors must contribute to the high rates of calf mortality seen in some years. In principle, chronic and roughly constant stresses caused by Kelp-Gull attacks could sensitize calves to these other factors, which otherwise might cause relatively little mortality. However, it is striking that only 20 calves died at Valdés in 2014, the year with the lowest gull attack frequency (19.2%) in Golfo Nuevo since 2006 (average frequency for 2005–2013 was 28%, [23], M. Sironi pers. obs.). Kelp Gulls attack newborn and juvenile Cape fur seals *Arctocephalus pusillus pusillus* in Namibia consuming their eyes, dermal layers of skin and other soft tissues which frequently can cause their death [41]. This suggests that Kelp-Gull attacks could potentially lead to the deaths of other young marine mammals under circumstances where they cannot easily recover from injuries caused by the gulls.

The intensity of wounding is currently higher in Golfo Nuevo than in Golfo San José. Solitary adults, juveniles and mother-calf pairs with lesions were rarely seen in Golfo Nuevo until 1986 [21]. We found that from the 1970s to the 1990s, larger numbers and areas of lesions were recorded on living mother-calf pairs sighted in Golfo San José, but by the 2000s more lesions were recorded in Golfo Nuevo. More lesions were also found on dead calves that stranded in Golfo Nuevo in the 2000s compared to those in Golfo San José. The increase in wounding in Golfo Nuevo seen here from retrospective analysis of aerial survey photos supports previous behavioral observations of higher gull attack frequencies in Golfo Nuevo compared to Golfo San José in the 2000s. From 2005 to 2013, the average frequency of attacks in Golfo Nuevo (28%) was almost 50% higher than in Golfo San José (19%) [23]. Interestingly, most (76%) calves that died at Valdés in 2003–2011 were found in Golfo Nuevo. This could result in part from a net movement of whales from Golfo San José to Nuevo, or from other habitat differences [16]. But it also coincides with higher attack frequencies and wounding in Golfo Nuevo ([23] and findings presented here).

Larger calves (living and dead) tend to have more and larger lesions than small calves. Most small living and dead calves without lesions were probably newborns. The increased wounding with calf length (~age) confirms Thomas's [20] observation that gull lesions on living mothers increased in size through the season. If calves have become the preferred targets of Kelp Gulls, then the longer they remain at Valdés, the greater may be the energetic costs they suffer, with potentially significant effects on survival.

Numbers and areas of lesions were not compared for living *versus* dead calves because there are many differences between the two samples. First, data collection occurs over different time frames, so the samples have different age distributions. Data collection for living calves occurs over a two-day period in September or early October when most calves are at least one month old. However, data collection for dead calves occurs from June through December, and includes all ages from newborns to calves nine meters in length. The majority (87%) of living calves that could be examined for gull lesions were large (≥50% their mother’s length), but only half (44%) of dead calves examined for gull lesions were large (≥6 meters). Second, the effort to count and record gull-inflicted lesions in dead calves increased since 2007, while the aerial survey effort to sample living whales has not changed much since the 1980s. Most necropsy reports previous to 2007 do not include enough detail about the number and size of gull lesions and few photographs were taken during external assessments. For example, lesions were not recorded for the majority of dead calves that stranded in 2003 and 2006. Third, a smaller proportion of dead calves (44%) than living calves (59%) were included in the gull lesion analyses presented here, owing mainly to their stranded position, state of decomposition, or in fewer cases, poor photo quality. The percentage is even smaller for number and area of lesions (~40%) for these same reasons. Thus, our findings about lesions on dead calves represent less than half of all the calves found dead at Valdés. These limitations cannot be overcome because dead whales are usually found in advanced states of decomposition and with major skin loss or damage [30].

The depths of lesions have been little studied, but could be an important factor determining the damage from gull attacks. In contrast to lesions on the epidermis, lesions that reach more deeply into highly vascularized layers such as the dermis or the hypodermis [42] are more likely to facilitate fluid loss. Most gull-inflicted lesions analyzed in dead right whales at Valdés were present in both the epidermis and dermis and a few penetrated to the underlying hypodermis (D. McAloose et al. submitted). When analyzing a sequence of aerial photographs of a living mother in 1999, we observed a Kelp Gull repeatedly gouge pieces of blubber from a lesion until it began to bleed. Unfortunately, we are not able to measure lesion depth in photographs of living whales, but since 2013, the SRWHMP has been taking three-dimensional measurements of wounds (number, size and depth) on dead calves. Further research is needed to improve our understanding of the severity of gull lesions in individual whales.

Evidence that similar types of parasite harassment can lead to fatigue and stress in mammals is accumulating for lions, and for wild and domestic ungulates. Die-offs of these other large mammals have been associated with biting by *Stomoxys* flies [43–45]. Kelp Gulls usually chase the whales by flying over them or resting on the water until they surface to breathe and then continue their attacks. Continuous harassment could potentially increase stress levels in mothers and calves and could negatively affect their body condition. Gull attacks on single mother-calf pairs can go on for more than an hour [21]. Further research is needed to evaluate whether gull attacks increase the whales’ metabolic stress and compromise their body condition and overall welfare.

Gull harassment could impact calf survivorship if calves invest more energy avoiding gull attacks and healing wounds than playing, resting and nursing. Swimming and play are the most frequent activities of undisturbed calves, and play may be critical for developing the strength and motor skills needed for migration to the feeding grounds [12]. Play may also be important for the development of adult behavior and social skills for survival and successful reproduction [12,46], as well as promoting general neural and muscular development in calves as described for other mammals [47,48].

Although mothers rest more than calves at Península Valdés [20], periodic rests may also help calves allocate energy to growth. Calf play is usually stopped by mothers, perhaps to conserve the energy of their young [12]. Both playing and resting increase with calf length [12], suggesting that they are essential to normal development. If Kelp-Gull attacks reduce the time spent in resting and swimming slowly by 25% and increase the time invested in swimming at faster speeds by almost fourfold [16], calves may be allocating more energy into fleeing gull attacks and thus diminishing the energy allocated to grow and develop normally which might compromise their welfare and overall survival.

Since the 1990s, mothers have learned to evade gull attacks but calves have not, and the numbers and sizes of lesions on calves have continued to increase. Kelp Gull harassment has clearly become a significant threat to the health and welfare of the Península Valdés right whale population. How best to mitigate that threat is not obvious, but a project to cull the Kelp Gull population at Valdés was initiated in 2012 by the provincial government of Chubut in collaboration with researchers from Centro Nacional Patagónico (CENPAT-CONICET). Continuing the long-term data series that were used here will greatly facilitate efforts to evaluate the effectiveness of this culling.

## Supporting Information

S1 Fig Gull-inflicted lesions on a whale silhouette. The drawing shows the total back area (TBA) that extends from the fat roll to the beginning of the tail stock and laterally to the "shoulders" (dotted line) and gull-inflicted lesions of different sizes (solid lines, circles).
(TIF)

S1 File Wounding of living and dead whales at Golfo San José and Golfo Nuevo. Mean and standard deviation of number and area of lesions on living mothers (a) and calves (b) (Table A) and on dead calves (Table B). Sample size is indicated between parentheses. In Table A, p-values (ANOVA) comparisons among gulfs are to the right of the number and area columns. Data were collected using aerial photographs in 1974–2011 for living mother-calf pairs, and from necropsy photographs of dead calves in 2003–2011.
(ZIP)
