# Correction: Increased Wounding of Southern Right Whale (*Eubalaena australis*) Calves by Kelp Gulls (*Larus dominicanus*) at Península Valdés, Argentina

**DOI:** 10.1371/journal.pone.0142969

**Published:** 2015-11-10

**Authors:** Carina F. Marón, Lucas Beltramino, Matías Di Martino, Andrea Chirife, Jon Seger, Marcela Uhart, Mariano Sironi, Victoria J. Rowntree

Fig 2 appears incorrectly in the published article. Please see the corrected version here.

**Fig 2:**
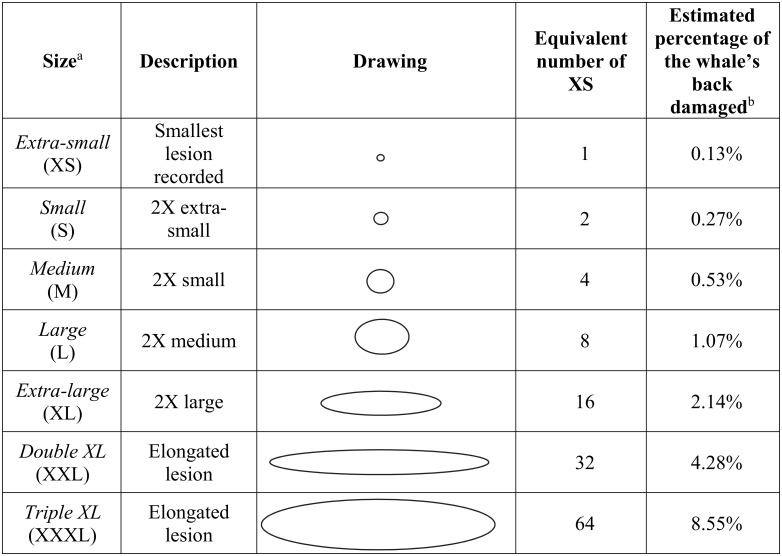
Sizes of gull lesions on southern right whales and their equivalent number of extra-small lesions. ^a^Abbreviations: Extra-small (XS), Small (S), Medium (M), Large (L), Extra-large (XL), Double XL (XXL) and Triple XL (XXXL). ^b^The total back area or TBA (100%) available for scoring extended from the fat roll (behind the blowholes) to the beginning of the tail stock and down the sides to the region of the back that is above the water or clearly visible through the water when the whale is close to the surface (see S1 Fig). See methods for a complete explanation.
