# Supplementary figures and images for: Increased Wounding of Southern Right Whale (Eubalaena australis) Calves by Kelp Gulls (Larus dominicanus) at Península Valdés, Argentina
Source: PLoS One. 2015 Oct 21;10(10):e0139291. doi: 10.1371/journal.pone.0139291 (PMC4619304; doi:10.1371/journal.pone.0139291)

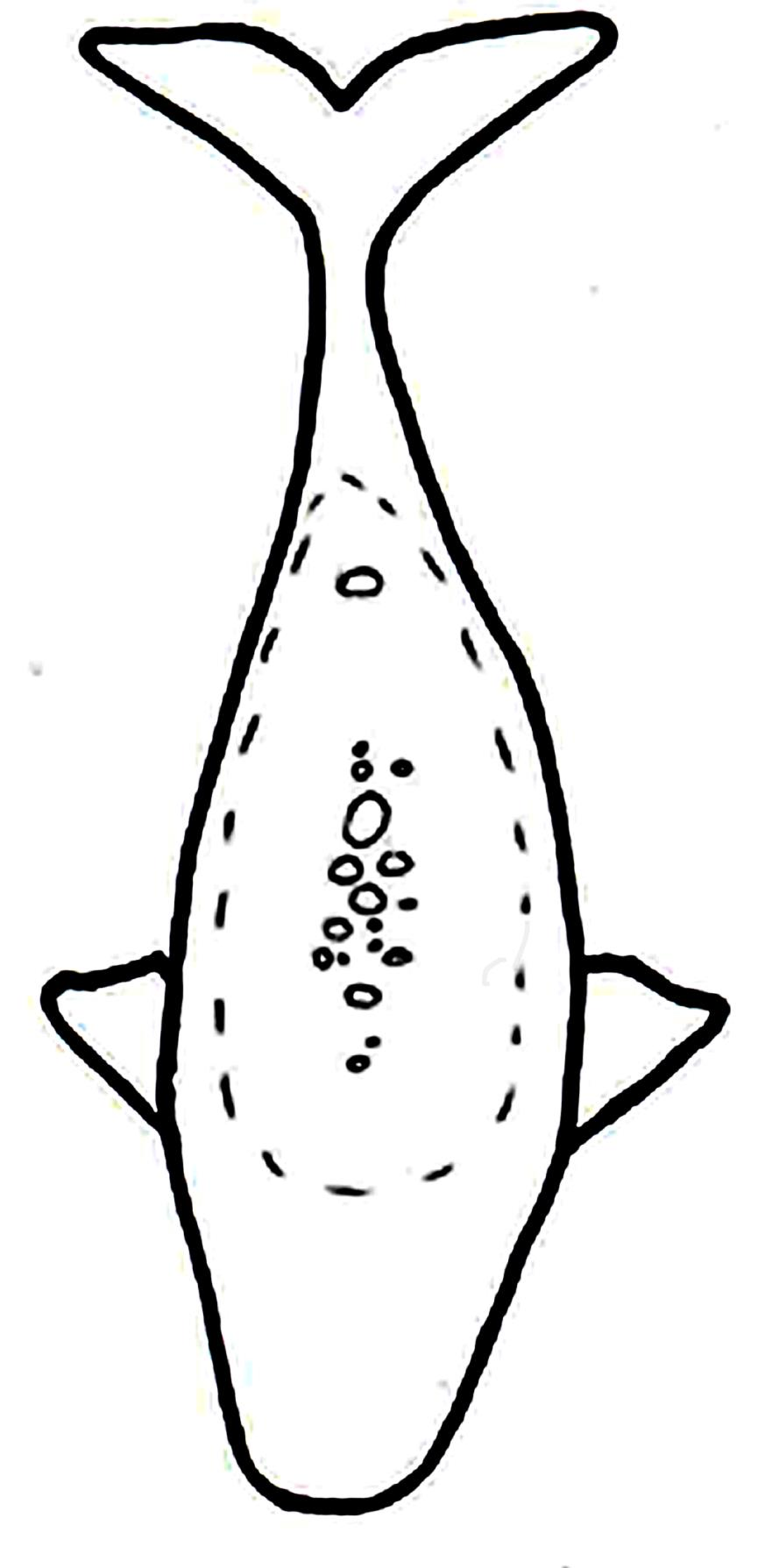

Supplement: S1 Fig — The drawing shows the total back area (TBA) that extends from the fat roll to the beginning of the tail stock and laterally to the "shoulders" (dotted line) and gull-inflicted lesions of different sizes (solid lines, circles). (TIF) [file pone.0139291.s001.tif]
