# Supplementary material for: Increased Wounding of Southern Right Whale (Eubalaena australis) Calves by Kelp Gulls (Larus dominicanus) at Península Valdés, Argentina
Source: PLoS One. 2015 Oct 21;10(10):e0139291. doi: 10.1371/journal.pone.0139291 (PMC4619304; doi:10.1371/journal.pone.0139291)
Supplement: S1 File — Mean and standard deviation of number and area of lesions on living mothers (a) and calves (b) (Table A) and on dead calves (Table B). Sample size is indicated between parentheses. In Table A, p-values (ANOVA) comparisons among gulfs are to the right of the number and area columns. Data were collected using aerial photographs in 1974–2011 for living mother-calf pairs, and from necropsy photographs of dead calves in 2003–2011. (ZIP) [file pone.0139291.s002.zip › Supporting Information/S1 Fig_C Maron.doc]

**S1 Fig. Gull-inflicted lesions on a whale silhouette.** The drawing shows the total back area (TBA) that extends from the fat roll to the beginning of the tail stock and laterally to the "shoulders" (dotted line) and gull-inflicted lesions of different sizes (solid lines, circles).
