# Supplementary material for: Increased Wounding of Southern Right Whale (Eubalaena australis) Calves by Kelp Gulls (Larus dominicanus) at Península Valdés, Argentina
Source: PLoS One. 2015 Oct 21;10(10):e0139291. doi: 10.1371/journal.pone.0139291 (PMC4619304; doi:10.1371/journal.pone.0139291)
Supplement: S1 File — Mean and standard deviation of number and area of lesions on living mothers (a) and calves (b) (Table A) and on dead calves (Table B). Sample size is indicated between parentheses. In Table A, p-values (ANOVA) comparisons among gulfs are to the right of the number and area columns. Data were collected using aerial photographs in 1974–2011 for living mother-calf pairs, and from necropsy photographs of dead calves in 2003–2011. (ZIP) [file pone.0139291.s002.zip › Supporting Information/S1 file_Supporting information_C Maron.doc]

**S1 File. Wounding of living and dead whales at Golfo San José and Golfo Nuevo.**

**Table A.**

a)

| **Decade** | **Mean number of lesions on living mothers** | | | **Mean area of lesions on living mothers** | | |
| --- | --- | --- | --- | --- | --- | --- |
|  | Golfo San José | Golfo Nuevo | p-value | Golfo San José | Golfo Nuevo | p-value |
| 1970s | 0.08±0.35 (n=39) | 0.00±0.00 (n=8) | 3.51x10^-06^ | 0.13±0.66 (n=39) | 0.00±0.00 (n=8) | 2.80x10^-06^ |
| 1980s | 4.18±3.53 (n=98) | 0.58±1.12 (n=159) | 2.00x10^-16^ | 6.64±5.42 (n=98) | 0.82±1.53 (n=159) | 2.00x10^-16^ |
| 1990s | 2.46±2.64 (n=39) | 0.84±1.12 (n=38) | 8.41x10^-04^ | 3.95±3.66 (n=39) | 1.45±2.05 (n=38) | 4.22x10^-04^ |
| 2000s | 3.38±1.98 (n=234) | 4.22±2.16 (n=242) | 1.07x10^-04^ | 5.71±4.53 (n=234) | 6.82±4.93 (n=242) | 0.01 |

b)

| **Decade** | **Mean number of lesions on living calves** | | | **Mean area of lesions on living mothers** | | |
| --- | --- | --- | --- | --- | --- | --- |
|  | Golfo San José | Golfo Nuevo | p-value | Golfo San José | Golfo Nuevo | p-value |
| 1970s | 0.14±0.74 (n=29) | 0.00±0.00 (n=6) | 7.59x10^-03^ | 0.28±1.49 (n=29) | 0.00±0.00 (n=6) | 0.01 |
| 1980s | 2.22±2.85 (n=73) | 0.15±0.77 (n=103) | 4.32x10^-11^ | 3.68±4.66 (n=73) | 0.18±0.87 (n=103) | 4.08x10^-12^ |
| 1990s | 1.93±2.31 (n=29) | 0.28±1.17 (n=32) | 7.31x10^-04^ | 3.24±3.73 (n=29) | 0.56±2.34 (n=32) | 1.24x10^-03^ |
| 2000s | 8.67±5.05 (n=178) | 9.57±4.98 (n=143) | 0.11 | 19.47±16.41 (n=178) | 20.15±14.77 (n=143) | 0.7 |

**Table B.**

| **Gulf** | **Lesions on dead calves** | |
| --- | --- | --- |
|  | Mean number | Mean area |
| Golfo San José | 1.85±3.18 (n=46) | 4.00±7.58 (n=43) |
| Golfo Nuevo | 4.13±4.64 (n=139) | 11.68±15.02 (n=133) |

Mean and standard deviation of number and area of lesions on living mothers (a) and calves (b) (Table A) and on dead calves (Table B). Sample size is indicated between parentheses. In Table A, p-values (ANOVA) comparisons among gulfs are to the right of the number and area columns. Data were collected using aerial photographs in 1974-2011 for living mother-calf pairs, and from necropsy photographs of dead calves in 2003-2011.
